# Results of three-year mass screening programme for lung cancer using mobile low-dose spiral computed tomography scanner

**DOI:** 10.1054/bjoc.2000.1531

**Published:** 2001-01-01

**Authors:** S Sone, F Li, Z-G Yang, T Honda, Y Maruyama, S Takashima, M Hasegawa, S Kawakami, K Kubo, M Haniuda, T Yamanda

**Affiliations:** Departments of Radiology, Laboratory Medicine, Internal Medicine and Surgery, Shinshu University School of Medicine; Telecommunications Advancement Organisation of Japan, Matsumoto Research Centre, Shinshu University School of Medicine, 3-1-1 Asahi, Matsumoto, 390-8621, Japan; JA Azumi General Hospital, 3207-1 Ikeda, Nagano, 399-8695, Japan

**Keywords:** lung cancer, screening, low-dose computed tomography (CT)

## Abstract

The aim of this study was to evaluate the usefulness of annual screening for lung cancer by low-dose computed tomography (CT) and the characteristics of identified lung cancers. Subjects consisted of 5483 general population aged 40–74 years, who received initial CT scans in 1996, followed by repeat annual scans for most subjects in 1997 and 1998, with a total of 13 786 scans taken during 1996–1998. Work-up examinations for patients with suspicious lesions were conducted using diagnostic CTs. The initial screening in 1996 detected suspicious nodules in 279 (5.1%) of 5483 subjects, and 22 (8%) were confirmed surgically to have lung cancer. Corresponding figures in 1997 and 1998 screening studies were 173 (3.9%) of 4425 and 25 (14%) of 173, and 136 (3.5%) of 3878 and 9 (7%) of 136, respectively. The sensitivity and specificity of detecting surgically confirmed lung cancer were 55% (22/40) and 95% (4960/5199) in 1996 and 83% (25/30) and 97% (4113/4252) in 1997 screening, respectively. 88% (55/60) of lung cancers identified on screening and surgically confirmed were AJCC stage IA. Our trial allowed detection of nearly 11 times the expected annual number of early lung cancers. Repeat CT allowed the detection of more aggressive, rapidly growing lung cancers, compared to those in the initial screening. © 2001 Cancer Research Campaign http://www.bjcancer.com


British Journal of Cancer (2001) 84(1), 25–32
© 2001 Cancer Research Campaign
doi: 10.1054/ bjoc.2000.1531 available online at http://www.idealibrary.com on IDEAL®

http://www.bjcancer.com

### Results of three-year mass screening programme for lung cancer using mobile low-dose spiral computed tomography scanner

**S Sone[1,5,6], F Li[1,5], Z-G Yang[1,5], T Honda[2], Y Maruyama[1], S Takashima[1], M Hasegawa[1], S Kawakami[1], K Kubo[3], M Haniuda[4] and T Yamanda[4]**

Departments of [1]Radiology, [2]Laboratory Medicine, [3]Internal Medicine and [4]Surgery, Shinshu University School of Medicine, and [5]Telecommunications Advancement Organisation of Japan, Matsumoto Research Centre, Shinshu University School of Medicine, 3-1-1 Asahi, Matsumoto, 390-8621 Japan; [6]JA Azumi General Hospital, 3207-1 Ikeda, Nagano, 399-8695, Japan (present address)

**Summary** The aim of this study was to evaluate the usefulness of annual screening for lung cancer by low-dose computed tomography (CT) and the characteristics of identified lung cancers. Subjects consisted of 5483 general population aged 40–74 years, who received initial CT scans in 1996, followed by repeat annual scans for most subjects in 1997 and 1998, with a total of 13 786 scans taken during 1996–1998. Work-up examinations for patients with suspicious lesions were conducted using diagnostic CTs. The initial screening in 1996 detected suspicious nodules in 279 (5.1%) of 5483 subjects, and 22 (8%) were confirmed surgically to have lung cancer. Corresponding figures in 1997 and 1998 screening studies were 173 (3.9%) of 4425 and 25 (14%) of 173, and 136 (3.5%) of 3878 and 9 (7%) of 136, respectively. The sensitivity and specificity of detecting surgically confirmed lung cancer were 55% (22/40) and 95% (4960/5199) in 1996 and 83% (25/30) and 97% (4113/4252) in 1997 screening, respectively. 88% (55/60) of lung cancers identified on screening and surgically confirmed were AJCC stage IA. Our trial allowed detection of nearly 11 times the expected annual number of early lung cancers. Repeat CT allowed the detection of more aggressive, rapidly growing lung cancers, compared to those in the initial screening. © 2001 Cancer Research Campaign http://www.bjcancer.com

**Keywords:** lung cancer; screening; low-dose computed tomography (CT)

Lung cancer is the primary cause of cancer death. The cure rates have remained low with only 10% of patients cured of this disease. According to Landis et al (1998) the estimated annual incidence of, and mortality from, lung cancer in the United States in 1998 were 171 500 and 160 100, respectively. The low cure rate is due to a combination of difficulty in detecting early stage disease and low cure rate for advanced disease. Mountain (1997) analysed the end results of 5230 patients treated for primary lung cancer. The results showed that stage I A cancer (TIN0M0: tumour with the greatest dimension ≤30 mm, no nodal involvement or distant metastases) represented 13% of the total population and had a 5-year survival rate of 61%. However, the majority of patients in that study had more advanced cancers with a more dismal outcome. Surgical treatment is expected to be associated with a fairly high 5-year survival rate for small lung cancers <20 mm without nodal involvement, or even higher rate for those <15 mm which are often free of nodal involvement (Sagawa et al, 1990; Oda et al, 1998). Although this is a difficult goal to achieve by conventional chest radiography, as was noted by Sone et al (2000), it is rather attainable using low-dose computed tomography (CT), as has been shown by Kaneko et al (1996), Sone et al (1998) and Henschke et al (1999).

We report here the results of annual repeat screenings for lung cancer using low-dose CT, with a particular focus on the performance of low-dose CT and characteristics of the detected lung cancers.

*Received 27 April 2000*
*Revised 31 August 2000*
*Accepted 13 September 2000*

*Correspondence to:* S Sone

### SUBJECTS AND METHODS

### Background

We conducted a population-based lung cancer screening trial from 1996 through 1998 as part of a protocol designed by Telecommunications Advancement Organisation (TAO) of Japan, Matsumoto Research Centre, as has been previously described by Sone et al (1998). The programme was limited to a 5-year period (1995–1999), which precluded long-term follow-up necessary to determine the final clinical outcome of the patients found in this screening. Further it was estimated that we could manage nearly 5000 to 10 000 participants with our mobile screening CT system in use. Under these limited conditions on the sample size and study duration, we could not aim to examine the possible benefit of screening for lung cancer based on a reduction in lung cancer mortality, we aimed to know among the general population the frequency of low-dose CT detected suspicious nodules, the results of work-up examinations together with the frequency of lung cancers among low-dose CT detected suspicious nodules, and characteristics of cancers found in the screening. While, according to Henschke et al (1994), in order to know the efficacy of screening for lung cancer with CT, we need to examine the three questions in a given type of the screenee: (1) probability of CT-detected small solitary pulmonary nodules (SSPN), (2) probability of malignancy, given detected SSPN, and (3) probability of resection, given malignancy of the SSPN and cure, given resection of SSPN; and, in order to determine the first question, which is an only specific concern to screening, we need to screen some 1000 persons at high-risk for lung cancer, with the expected yield of 100

to 150 detected SSPNs from these screenees. A much larger series of SSPN is required to study the second question, the prevalence of malignancy among the detected SSPNs, by getting an appreciable number of malignant SSPN, because the prevalence of malignant SSPN among high-risk population is estimated to be of the order of 1%. We defined our sample size of 5000 to 10 000, which was fairly larger than that indicated by Henschke et al, because we aimed to conduct our trial of CT screening for lung cancer using CT among the general population, which included never-smoked inhabitants in this rural area in Japan. In our study, inhabitants of 29 local municipalities in the Nagano Prefecture, Japan, who were 40 years of age or older were specifically requested in 1996 to volunteer for this programme. The annual mortality rate due to lung cancer in the same region was 37.3 per 100 000 population in 1998. Therefore, it was expected in our trial at least nearly 100 SSPNs be detected at each annual screening to undergo work-up examination, which would permit us to estimate the probability of CT-detected SSPN. Although we expected to encounter at least several cases with lung cancer among the total 5000 to 10 000 participants, we were not sure about the expected number of cancer cases because no information on this aspect was available. All subjects gave informed consent to receive base line and annual repeat CT scans of the thorax.

### Subjects

The material of this study comprised a total of 13 786 CT examinations, including 5483 initial CT scans in 1996, 4425 first-year repeat CT scans in 1997 and 3878 second-year repeat CT scans in 1998 (Table 1). We recommended that all participants receive the initial and two annual repeat screenings, however 1058 did not undergo the first annual repeat screening, which was conducted 11–13 months after the 1996 initial screening; these included 34 who had been found to have highly suspicious lung lesions at the initial screening and had undergone surgery (29) or were under follow-up observations (5), 113 who were followed up for probably benign lesions, and 911 who did not receive it for reasons of their own.

Among the total of 3878 subjects who received the second repeat CT scans in 1998, 3522 received the 3 screenings from 1996 through 1998, and 356 received the screening in 1996 but did

**Table 1** Subjects of annual screening by low-dose CT from 1996 to 1998

| | Initial screen (1996) | Annual repeat screening (1997) | Annual repeat screening (1998) | Total (1996–98) |
|---|---|---|---|---|
| Participants | 5483 | 4425 | 3878 | 13 786 |
| | | (Women / Men) | | |
| Gender | 2412 / 2971 | 2053 / 2373 | 1816 / 2062 | 6381 / 7405 |
| Mean age(years) | 64 | 66 | 63 | 65 |
| Age of never-smokers | | (Women / Men) | | |
| 40–44 (years in age) | 87 | 72 | 60 | 219 |
| | (76 / 11) | (59 / 13) | (48 / 12) | (183 / 36) |
| 45–49 | 151 | 121 | 104 | 376 |
| | (122 / 29) | (101 / 20) | (87 / 17) | (310 / 66) |
| 50–54 | 323 | 276 | 226 | 825 |
| | (265 / 58) | (232 / 44) | (196 / 30) | (693 / 132) |
| 55–59 | 448 | 373 | 315 | 1136 |
| | (375 / 73) | (308 / 65) | (264 / 51) | (947 / 189) |
| 60–64 | 698 | 570 | 519 | 1787 |
| | (539 / 159) | (446 / 124) | (407 / 112) | (1392 / 395) |
| 65–69 | 776 | 635 | 580 | 1991 |
| | (600 / 176) | (494 / 141) | (448 / 132) | (1542 / 449) |
| 70–74 | 471 | 366 | 320 | 1157 |
| | (365 / 106) | (292 / 74) | (253 / 67) | (910 / 247) |
| Subtotal | 2954 | 2413 | 2124 | 7491 |
| | (2342 / 612) | (1932 / 481) | (1703 / 421) | (5977 / 1514) |
| Age of smokers (>1 pack-year) | | (Women / Men) | | |
| 40–44 (years in age) | 150 | 116 | 105 | 371 |
| | (15 / 135) | (13 / 103) | (12 / 93) | (40 / 331) |
| 45–49 | 195 | 141 | 122 | 458 |
| | (17 / 178) | (11 / 130) | (11 / 111) | (39 / 419) |
| 50–54 | 266 | 207 | 173 | 646 |
| | (38 / 228) | (29 / 178) | (25 / 148) | (92 / 554) |
| 55–59 | 301 | 222 | 188 | 711 |
| | (24 / 277) | (16 / 206) | (17 / 171) | (57 / 654) |
| 60–64 | 474 | 374 | 340 | 1188 |
| | (25 / 449) | (17 / 357) | (13 / 327) | (55 / 1133) |
| 65–69 | 667 | 554 | 486 | 1707 |
| | (34 / 633) | (21 / 533) | (24 / 462) | (79 / 1628) |
| 70–74 | 476 | 398 | 340 | 1214 |
| | (17 / 459) | (14 / 384) | (11 / 329) | (42 / 1172) |
| Subtotal | 2529 | 2012 | 1754 | 6295 |
| | (170 / 2359) | (121 / 1891) | (113 / 1641) | (404 / 5891) |
| | | (Never-smoker / Smoker > 1 pack-year) | | |
| Smoking in total | 2954 / 2529 | 2413 / 2012 | 2124 / 1754 | 7491 / 6295 |
| in women | 2342 / 170 | 1932 / 121 | 1703 / 113 | 5977 / 404 |
| in men | 612 / 2359 | 481 / 1891 | 421 / 1641 | 1514 / 5891 |

 *© 2001 Cancer Research Campaign*

not receive it in 1997 for reasons of their own. Consequently, among the total of 5483 subjects screened in 1996, 3522 were further screened twice (1997 and 1998), 903 further screened once in 1997, 356 were further screened only in 1998, and 702 received no repeat screening.

Among the total of 13 786 scans, 6381 (46%) were conducted in women and 7405 (54%) were in men. With respect to smoking habits, 7491 (54%) were never-smokers, and 6295 (46%) smokers (>1 pack year, the average number of packs of cigarettes smoked per day × duration of smoking in years). Among the 6381 examined females, 5977 (94%) were never-smokers, while among the 7405 examined males, 1514 (20%) were never-smokers (Table 1).

The median age at the initial screening for the screenees was 63 years for women and 64 years for men. The distribution of screenees according to age and sex was as follows; age, 40–44 years: 223 (2%) women and 367 (3%) men, 45–49 years: 349 (3%) women and 485 (4%) men, 50–54 years: 785 (6%) women and 686 (5%) men, 55–59 years: 1004 (7%) women and 843 (6%) men, 60–64 years: 1447 (10%) women and 1528 (11%) men, 65–69 years: 1621 (12%) women and 2077 (15%) men, and 70–74 years: 952 (7%) women and 1419 (10%) men (Table 1).

### Methods

### *Screening CT scans and interpretation*

All participants underwent a low-dose non-enhanced spiral CT scan of the thorax in a mobile CT unit (Model CT-W950SR; Hitachi Medical, Tokyo, Japan) free-of-charge. The scan parameters were 120 kVp, 50 mA in 1996 and 25 mA in 1997 and 1998, 10 mm collimation, 10 mm $s^{-1}$ table speed, 2 s rotation of the X-ray tube, and pitch of 2. Images were displayed and interpreted on two monochrome high-resolution cathode-ray tubes (CRT) at 3 display conditions to adequately examine the lungs, hilar bronchi and mediastinum (width 1000 HU, level −700 HU; width 1500 HU, level −550 HU; and width 300 HU, level 20 HU, respectively). The chest radiologists interpreted annual repeat CT images to detect any abnormality, and then comparison interpretations were done to know the interval changes of the lesion, shown on the images of the repeat screening and initial screening. Interpretation of the images was based on commonly used morphologic and density characteristics and interval changes of the lesion. We aimed to classify any lesion into one of 7 categories, as follows: (A) unsatisfactory examination, (B) normal, (C) lung abnormality of little clinical importance, (D) non-cancerous lung lesion, (Ed) non-cancerous but suspicious lung lesion, (E) suspicion of lung cancer, (F) small lung nodule (<3 mm in diameter). The results of interpretation were conveyed to each participant via health professionals at each municipality.

### *Diagnostic work-up examinations*

A complete diagnostic work-up examination with conventional chest radiographs (usually only by the frontal radiograph) and diagnostic CT (including thin-section, high-resolution CT, HRCT) was recommended for those individuals with CTs classified as categories Ed, E or F, and most of them underwent work-up examinations at our hospital. Patients classified as category D were recommended to undergo work-up examinations at the local hospitals. In our hospital, diagnostic CT scans were performed with a state-of-the-art CT scanner (HiSpeed Advantage, GE Medical Systems, Milwaukee, WI). Contiguous 10 mm sections for the whole lung and thin section images (1 mm collimation) to cover the entire nodule in question were taken and interpreted based on the morphologic and density characteristics and the presence of growth tendency of the lesion. Magnetic resonance imaging or transbronchial lung biopsy was also performed, when necessary. For subjects with indeterminate lesions, serial follow-up CTs were conducted at 3, 6, 12, 18 and 30 months, and surgery was recommended when any increase in size or density of the nodule was noted. The results of the work-up studies were conveyed to the patient; our explanation included the results of the initial and annual repeat screening CT images, the corresponding diagnostic CT images, as well as the interval changes and probable CT diagnosis of the detected lung lesion.

### Data analysis

We compared the initial and annual repeat CT screenings for the proportions of lung nodules detected on screening CT and surgically confirmed lung cancers among CT detected suspicious nodules, and the characteristics of the patients with surgically confirmed lung cancers. Disease staging was determined histopathologically according to the TNM (tumour, nodal involvement, metastasis) classification system of the American Joint Committee on Cancer (AJCC) and Union Internationale Contre le Cancer’s (UICC) in 1996.

### RESULTS

### Frequency of suspicious nodules detected on screening CTs (Table 2)

In the initial CT screening in 1996, 5.1% (279/5483) (95% confidence interval (CI), 2.5–7.7) had possible or probable cancer or indeterminate nodules < 3 mm in diameter. In comparison, in the annual repeat CTs in 1997 and 1998, 3.9% (173/4425, CI, 1.0–6.8) and 3.5% (136/3878, CI, 0.4–6.7), had suspicious lesions, respectively. A careful comparison between the CTs of the year and the preceding year(s) helped to reduce the number of patients, who had stable lesions, to be classified into category Ed, E or F.

**Table 2** Low-dose CT detected suspicious nodular lesions during 1996 to 1998

| | Initial screening | Annual repeat screening | | Total |
|---|---|---|---|---|
| | (1996) | (1997) | (1998) | (1996–98) |
| Number of screenees | 5483 (100%) | 4425 (100%) | 3878 (100%) | 13 786 (100%) |
| Probable cancer (E) | 109 (2.0%) | 40 (0.9%) | 26 (0.7%) | 175 (1.3%) |
| Possible cancer (Ed) | 61 (1.1%) | 131 (3.0%) | 110 (2.8%) | 302 (2.2%) |
| Small nodule, <3 mm (F) | 109 (2.0%) | 2 (–) | – | 111 (0.8%) |
| Subtotal (Ed, E and F) | 279 (5.1%) | 173 (3.9%) | 136 (3.5%) | 588 (4.3%) |
| Probable benign (D) | 397 (7.2%) | 100 (2.3%) | 109 (2.8%) | 606 (4.4%) |

© 2001 Cancer Research Campaign

**Table 3** Frequency of lung cancers among low-dose CT-detected suspicious nodules

| | Initial screening | | | Annual repeat screening | | | | | | Total | | |
|---|---|---|---|---|---|---|---|---|---|---|---|---|
| | | (1996) | | | (1997) | | | (1998) | | | (1996–98) | |
| Screenees | | 5483 | | | 4425 | | | 3878 | | | 13 786 | |
| Work-ups recommended | | 279 | | | 173 | | | 136 | | | 588 | |
| done | | 266 | | | 168 | | | 129 | | | 563 | |
| Cancer, highly suspicious | | 34 | | | 32 | | | 16 | | | 82 | |
| Surgery done | 29 | | | 30 | | | 13 | | | 72 | | |
| (histology) | | | | | | | | | | | | |
| Cancer | | | 22 | | | 25 | | | 9 | | | 56 |
| AAH* | | | 2 | | | 4 | | | 3 | | | 9 |
| Non-ca | | | 5 | | | 1 | | | 1 | | | 7 |
| Postponed | 2 | | | 1**, 1 | | | 1 | | | 5 | | |
| Rejected | 3 | | | | | – | 2 | | | 5 | | |
| Indeterminate | | – | | | 2 | | | 1 | | | 3 | |
| Non-cancerous | | 232 | | | 134 | | | 112 | | | 478 | |
| False negative(1)# | | 1 | | | – | | | – | | | 1 | |
| False negative(2)## | | – | | | 2 | | | 1 | | | 3 | |
| False negative(3)### | | (17) | | | (3) | | | (?) | | | (20 + ?) | |
| Cancer patients confirmed | 23 | | | 27 | | | 10 | | | 60 | | |
| CT-detected, surgically confirmed cancers | 22 | | | 25 | | | 9 | | | 56 | | |
| (per 100 000 population) | | (401) | | | (565) | | | (232) | | | (406) | |

*: atypical adenomatous hyperplasia. **: small cell lung cancer, confirmed by transbronchial biopsy, and chemotherapy was done. (1)#: small cancer of hilar type detected by sputum cytologic exam. (2)##: cancer detected from the categories other than the suspicious nodules, e.g. E (probable cancer), Ed (possible cancer) or F (small nodule, <3 mm). (3)###: cancer > 5 mm in size, that is retrospectively visible, but was only detected in a screening of the one year later (or, occasionally two years later).

### Frequency of lung cancers among suspicious nodules detected on screening CTs (Table 3)

Of the 279 participants who had suspicious nodules detected on 1996 screening, 266 (94%) underwent further radiographic evaluation. Such tests allowed the classification of 34 cases with highly suspicious cancer nodules. Among these, 29 underwent surgery, and 22 were histopathologically confirmed to have lung cancer (0.40% (CI, 0.23–0.57) of all those screened in 1996, or 7.9% (CI, 4.7–11.1) of 279 suspicious examinations at screening CT), 2 had atypical adenomatous hyperplasias (AAH), and 5 miscellaneous non-cancerous lesions. Surgery was postponed in two patients who had multiple small suspicious nodules found at work-up examinations and remain to date under follow-up observation, with the intention to perform surgery when any lesion shows a significant increase in size. 3 patients refused surgery. The diagnosis in the remaining 232 was non-neoplastic pulmonary lesions. There was one false negative finding on screening CT, which was detected by cytologic examination of the sputum, verified by bronchoscopy and treated by surgery.

Among 173 examinations with suspicious lesions at the first annual repeat CT of 1997, 168 (97%) received work-up CT examinations. 32 were diagnosed to have a highly suspicious cancer, 30 were subsequently treated surgically and 25 were confirmed lung cancer cases (detection rate: 0.56%, CI, 0.34–0.78, of the annual re-examinations of 4425, or 14.5%, CI, 9.1–19.9, of 173 suspected cases on repeat screening), 4 had atypical adenomatous hyperplasias (AAH), and one had non-cancerous lesion.

Among 136 CTs with suspicious lesions at the second annual repeat CT of 1998, 129 (95%) received work-up CT examinations, 16 were later diagnosed to have a highly suspicious cancer, 13 were subsequently treated surgically and 9 had lung cancer (detection rate: 0.23%, CI, 0.08–0.38, of the annual re-examinations of 3878, or 6.4%, CI, 2.2–10.6 of 136 suspected cases on repeat screening CT), 3 had atypical adenomatous hyperplasias (AAH), and one had non-cancerous lesion.

### Sensitivity and specificity of interpretation of screening CT and work-up examinations

Further examination of the cases detected in the repeat CT screening showed that among the 25 patients detected in 1997, 16 were retrospectively visible (as lesion >5 mm in diameter) in 1996; and among the 9 patients detected in 1998, one was visible in 1996 and 3 were visible in 1997. Therefore, we had 17 false negative readings, in addition to one negative reading for a cytologically detected cancer, in 1996; and 3 false negative cases and 2 additional negative cases due to error in classification in 1997, for the surgically confirmed cancer lesions >5 mm in diameter. Accordingly, the sensitivity was 55% (22/40 = 22 + 17 + 1) in 1996 and 83% (25/30 = 25 + 3 + 2) in 1997. We do not have reliable data regarding the number of false negative readings in 1998, because the third year annual repeat screening in 1999 was limited to those participants living in the city area.

As to the specificity of our interpretation of screening CTs, there were 60 cancer cases in 1996–1998, therefore 13 726 (13 786 − 60) negative CT examinations were performed, which were not related to the surgically verified lung cancer, and we made 13 198 negative readings (Interpretation Classifications of B, C and D); resulting in a specificity of 97% (13 198/13 726). The specificity was 95% in 1996, 97% in 1997 and 97% in 1998, respectively (details are omitted here).

### Characteristics of surgically confirmed lung cancers (Table 4)

Table 4–1 shows the characteristics of 60 patients with lung cancer by age, gender, smoking history and visibility of tumours on the

 © 2001 Cancer Research Campaign

**Table 4–1** Characteristics of 60 patients with CT-screening detected and surgically confirmed lung cancers (excluding AAH*)

| | Initial screen | Annual repeat screening | | Total |
|---|---|---|---|---|
| | (1996) | (1997) | (1998) | (1996–98) |
| Participants | 5483 | 4425 | 3878 | 13 786 |
| Cancer detected | 23 | 27 | 10 | 60 |
| Mean age (years) | 64 | 66 | 63 | 65 |
| Age of patients | | (Female / Male) | | |
| 40–44 (years) | – | – | – | – |
| 45–49 | 1/1 | 2/– | – | 3/1 |
| 50–54 | 1/– | – | –/1 | 1/1 |
| 55–59 | 1/3 | –/2 | 1/– | 2/5 |
| 60–64 | 3/1 | 2/4 | 1/3 | 6/8 |
| 65–69 | 2/3 | 4/5 | 2/1 | 8/9 |
| 70–74 | 3/4 | 4/4 | –/1 | 7/9 |
| Subtotal by gender | 11/12 | 12/15 | 4/6 | 27/33 |
| Smoking history | | (Never-smoker / Smoker > 1 pack-year) | | |
| 40–44 (years) | – | – | – | – |
| 45–49 | 1/1 | 2/– | – | 3/1 |
| 50–54 | 1/– | – | –/1 | 1/1 |
| 55–59 | 1/3 | –/2 | 1/– | 2/5 |
| 60–64 | 4/– | 4/2 | 1/2 | 9/4 |
| 65–69 | 2/3 | 4/5 | 2/1 | 8/9 |
| 70–74 | 4/3 | 4/4 | –/2 | 8/9 |
| Subtotal by smoking | 13/10 | 14/13 | 4/6 | 31/29 |
| Chest radiograph negative | 14(61%) | 19(70%) | 7(70%) | 40(67%) |

*: atypical adenomatous hyperplasia.

**Table 4–2** Characteristics of 60 patients with CT-screening detected and surgically confirmed lung cancers (excluding AAH*)

| | Initial screen | Annual repeat screening | | Total |
|---|---|---|---|---|
| | (1996) | (1997) | (1998) | (1996–98) |
| Cancer detected | 23 | 27 | 10 | 60 |
| Tumour size | | | | |
| <10 (mm) | 6 | 10 | 5 | 21 |
| 11–15 | 8 | 12 | 3 | 23 |
| 16–20 | 7 | 5 | 1 | 13 |
| 21–47 | 2 | – | 1 | 3 |
| Mean/median | 15.1/13.5 | 12.1/12.0 | 12.0/10.3 | 13.4/12.5 |
| Stage(pathologic) | | | | |
| 1A | 21 | 24 | 8 | 53 |
| 1B | 2 | – | – | 2 |
| 2A | – | – | 1 | 1 |
| 2B | – | 1 | – | 1 |
| 3A | – | 1 | – | 1 |
| 3B | – | 1 | – | 1 |
| 4 | – | – | 1 | 1 |
| Outcome of patients | | | | |
| Disease free | 21 | 21 | 10 | 55 |
| Death, due to lung cancer | 1 | 1 | – | 2 |
| Death, due to other than lung cancer | 1 | 2 | – | 3 |
| Histology (never-/smoker) | | | | |
| Adenoca (BAC**, well differentiated) | 16(12/4) | 18(12/6) | 8(4/4) | 42(28/14) |
| Adenoca (moderately-, poorly-differ) | 3(–/3) | 6(2/4) | – | 9(2/7) |
| Squamous | 4(1/3) | 1(–/1) | 1(–/1) | 6(1/5) |
| Small cell | – | 2(–/2) | 1(–/1) | 3(–/3) |

*: atypical adenomatous hyperplasia.
**: bronchioloalveolar cell carcinoma.

chest radiograph. Of these patients, none was younger than 45 years, 4 cancers (7%) were 45–49 years old, 2 (3%) were 50–54 years old, and the remaining 54 (90%) were 55–74 years old.

The incidence in females was 0.44% (11/2512) [CI, 0.18–0.70], which was slightly higher than 0.40% (12/2971) [CI, 0.17–0.63] in men, in the initial screening, and fewer women with a positive CT were identified in the repeat screening than men, particularly in the second repeat screening; 0.22% (4/1816) [CI, 0.01–0.43] in women vs. 0.29% (6/2062) [CI, 0.06–0.52] in men.

The proportion of patients with lung cancer in never-smokers was 0.44% (13/2954) [CI, 0.11–0.67], which was slightly higher than 0.40% (10/2529) [CI, 0.15–0.65] in smokers, in the initial screening; and it became slightly lower for never-smokers than for smokers at repeat CT, particularly in the second repeat screening:

© 2001 Cancer Research Campaign 

0.19% (4/2124) [CI, 0.01–0.37] in never-smokers vs. 0.34% (6/1754) [CI, 0.07–0.61] in smokers.

Two thirds (67%, 40/60 cases) of cancers detected on the low-dose CT were invisible by a retrospective interpretation study on the chest radiograph.

Anatomically, 41 (68%) lesions were detected in the right lung, and 19 (32%) in the left lung. The most common location of lung cancer was the right upper lobe (22 lesions, 37%), followed by right lower lobe (15 lesions, 25%), left lower lobe (11 lesions, 18%), left upper lobe (8 lesions, 13%) and right middle lobe (4 lesions, 7%).

Table 4–2 shows the characteristics of 60 patients with surgically verified lung cancer by size, stage, outcome and histology. The diameter of the tumours measured at diagnostic CT ranged from 4 to 47 mm (mean, 13.4 mm). Specifically, 44 (73%) measured less than 15 mm; 13 (22%) measured 16–20 mm; and 3 (5%) were >21 mm in diameter. 53 patients (88% (78.2–97.8)) were classified as AJCC Stage 1A (postsurgical staging). Postoperative follow-up (duration: 1.2–3.7 years) of the 60 patients showed 2 deaths due to lung cancer and 3 deaths due to causes unrelated to lung cancer. The present status of the remaining 55 patients verified individually within the last 2 months is still disease-free.

The most frequent cell type was adenocarcinoma; 51 (85%) were adenocarcinomas, 6 (10%) squamous cell carcinomas and 3 (5%) small cell carcinomas. Histologic type of cancer was different between never-smokers and smokers. The proportion of patients with bronchioloalveolar cell carcinoma (BAC) or well-differentiated adenocarcinoma in never-smokers was 90% (28/31 patients), which was markedly higher than 48% (14/29) in smokers. There was a close association between absence of smoking history and histologic diagnosis of BAC or well-differentiated adenocarcinoma ($\chi^2$ test of association, $P < 0.001$).

### DISCUSSION

The present report is based on the extended observations performed on two additional annual repeat CT screening programmes, following the initial results obtained in 1996 (12). Our study indicates that low-dose CT is an excellent imaging test for early detection of lung cancers measuring approximately 5 to 20 mm in size. It allowed detection of a large number of surgically confirmed cancer cases (56/13 786 CTs), representing a detection rate of 0.41%, or 406 per 100 000 subjects, which was nearly 11 times as high as the annual mortality rate from lung cancer (37.3 per 100 000) in this country area of Japan. A report released by the Japanese Research Committee of Studies on Evaluation of Effectiveness of Cancer Screening (Japanese publication, 1998) indicated that in 1995 nearly 6 700 000 Japanese of at least 40 years of age and heavy smoking high-risk group were screened through standard mass screening using chest radiograph and sputum cytologic examination. Among these, work-up examinations were required on nearly 170 000 subjects (2.5%), and 3144 (1.9%) were subsequently diagnosed with lung cancer (i.e., a detection rate of nearly 0.05% among all screenees). The results of our screening programme using low-dose CT differed in several aspects from the above study. (i) The detection rate of surgically verified patients with lung cancer was 0.41% (56/13 786); nearly 8 times the rate of the above programme using standard mass screening. (ii) The rate of work-up examination in our study was 4.3% (588/13 786), which was slightly higher than 2.5% with standard mass screening. (iii) The rate of identification of lung cancer among those who underwent work-up examinations due to suspected lesions to indeterminate abnormality was 10% (56/563), which was markedly higher than 1.9% in standard mass screening. (iv) Finally, our analysis showed that the proportion of cases with stage IA malignancy among cancers detected by CT screening was approximately 88% (53/60), which was markedly higher than those reported for stage I from Japan and USA, e.g. 51% (103/206) by Soda et al (1993) and 63% (44/70) by Flehinger et al (1999).

Kaneko et al (1996) reported an overall cancer detection rate of 0.43% in CT examinations conducted on men aged 50 years or older who were heavy smokers, >20 pack-year, and 93% of these cancers were stage I. Henschke et al (1999) conducted baseline low-dose CT on 1000 symptom-free volunteers, aged 60 years or more, with smoking habit of at least 10 cigarette pack-years. They were able to detect solitary non-calcified nodules in 23% of the subjects, and 12% of these were later confirmed to be malignant. The rate (12%) of malignancy among CT detected nodules was similar to 10% found in our study.

Many small cancers were detected in the initial CT screening in 1996, however, more cases were detected in the first annual repeat CT screening in 1997. This was probably due to improved interpretation gained after the first year. This was possible firstly by learning and experience gained by the radiologists with respect to characterization of small lung cancers, and secondly by the comparison interpretation system devised for interpreting repeat CT images. In our system, the new and previous CT images were displayed side by side on two CRT monitors, which enhanced the detection of trivial changes in the repeat CT image.

As to the characteristics of patients identified, we found as many cases of lung cancer in women as in men in the initial screening; the majority of women in this study were never-smokers and had mostly bronchioloalveolar carcinoma, BAC, or well differentiated adenocarcinoma, but the proportion of these histological types decreased in the repeat screening. This observation suggests the presence of a relatively large number of slowly growing cancers among the prevalence cases, particularly among never-smokers, and that never-smokers should be included in any screening programme, at least in the initial screening. However, we found fewer cases in never-smokers in the repeat screening; this was particularly evident in the third year, when the incidence cases appeared to form a large proportion of identified cancers, which tended to accompany small cell lung carcinoma, squamous cell carcinoma, and moderately to poorly differentiated adenocarcinomas. Because this group of cancers may grow rapidly, smokers should be screened at least once a year. On the contrary, although persons who are older than 55 years should be advised to receive CT screening in order to identify the majority of lung cancers at an early stage, never-smokers may better undergo it less frequently than smokers, probably once every 3 to 4 years. However, the appropriate frequency of CT screening for never-smokers should be determined in a larger data, which could be collected, for example, through multi-institutional co-operative studies.

At the time of preparation of this report, the mortality rates due to lung cancer among diagnosed and surgically treated (i.e., histologically confirmed) patients in this study are 4% (1/23) for the initial CT and 4% (1/27) for the first annual repeat CT. However, a longer follow-up study is necessary before any firm evaluation could be made of the outcome of small lung cancers detected in this study.

© 2001 Cancer Research Campaign

In our study, surgically treated and histologically confirmed lung cancers were limited to 56 cases (10%) of 588 suspicious lesions; this indicates that various types of pulmonary nodules, mostly non-cancerous, may be detected on low-dose screening CT and classified as suspicious lesions. Therefore, there is a real need for the development of an effective strategy for a correct diagnosis of malignant tumours among suspicious lesions found at screening, to allow proper management and selection of appropriate treatment with little delay in cases with lung cancer.

Although CT mass screening for lung cancer appears very promising for the detection of lung cancer mostly at the surgically curable stage, a careful evaluation of the benefits–risks and cost-effectiveness issues is important. According to Nishizawa et al (1996), the effective dose using low-dose CT scan of the chest is 3.6 mSv and the surface dose is 7.6 mGy, when CT scans are performed with a tube current set at 50 mA. Their risks–benefits analysis indicated that the benefits of annual CT screening for lung cancer outweigh the risk in men aged 40 years and women aged 45 years.

The economic aspects of screening programmes have been fully discussed by Eddy (1981). Our present programme did not provide sufficient data to allow a proper evaluation of the economic aspects of cancer screening. However, we tentatively calculated the cost of screening by CT per person to be at $50 (5000 Japanese yen), based on our 3-year CT screening programme (Asakura et al, Japanese publication, 1999). This was determined by taking into account the annual costs of capital equipment and maintenance costs of the mobile CT scanner, digital image display/archive unit, and reporting system, with depreciation time estimated at 5 years, in addition to labour costs, and the total of workdays estimated at 150 days per year and 100 participants tested a day. However, it is hoped that the cost of each test is reduced by the development of more economical and efficient systems designed mainly for screening chest diseases.

Asakura et al (Japanese publication, 1999) examined further the cost per person year saved, as is partly shown in Table 5–1. It costs more to save a person year of younger subjects, particularly in women, and it costs, for example, approximately $21296 for women and $8148 for men, respectively, to save a person year of 55–59 year-old subjects, when we compute the cost on the assumption that the detection rate by CT is the same as incidence rates and the other relevant data could be based on those obtained at our first 3 years annual low-dose CT screening for lung cancer. However, in reality, as is shown in Table 5–2, the detection rate for lung cancers in our screening by CT was much higher than the annual incidence rate; 10 to 15 times higher in female never-smokers and 2 to 15 times higher in male smokers, compared to the annual mortality rates. Consequently, we could expect to save a person year at a much lower cost by CT screening, at least in the initial screening. For example, the estimated costs could be approximately at $2290 ($21296/9.3 (= 211 of CT screening detected cases / 22.6 of age specific mortality rate)) for women and $728 [$8148/11.2 (=765/68.6) for men, respectively, to save a person year of the 55–59 year-old subject by CT screening.

The limitations of the present study include the small number of cases detected with lung cancers and a limited follow-up period of screening detected cancer patients to determine survival rates. These limits do not allow accurate definition of the optimal size of lung cancer to be detected, necessary for a favourable prognosis, which consequently limits determination of the appropriate frequency of CT screening, definition of subjects to be screened, and work-up examinations for patients with a positive screening CT scan.

**Table 5–2** Detection of lung cancer by low-dose CT

| Age (year) | Detection rates of lung cancer per 100 000 by CT screening in 1996–98 (Number of cancers/Number of participants) | | | |
|---|---|---|---|---|
| | Never-smoker | | Smoker | |
| | Female | Male | F | M |
| 40–44 | – (–/183) | – (–/36) | – (–/40) | – (–/331) |
| 45–49 | 968 (3/310) | – (–/66) | – (–/39) | 239 (1/419) |
| 50–54 | 144 (1/693) | – (–/132) | – (–/92) | 181 (1/554) |
| 55–59 | 211 (2/947) | – (–/189) | – (–/57) | 765 (5/654) |
| 60–64 | 431 (6/1392) | 759 (3/395) | – (–/55) | 353 (4/1133) |
| 65–69 | 519 (8/1542) | – (–/449) | – (–/79) | 553 (9/1628) |
| 70–74 | 769 (7/910) | 405 (1/247) | – (–/42) | 768 (9/1172) |
| Subtotal | 418 (27/5977) | 264 (4/1514) | – (–/404) | 492 (29/5891) |
| Subtotal | 414 (31/7491) | | 461 (29/6295) | |
| Total | 435(60/13 786) | | | |

**Table 5–1** Annual mortality rates by lung cancer by age, and cost to gain a person year by screening

| Age (year) | Annual mortality rates by lung cancer/100 000 Japanese in 1990 | | Cost ($) per person year gained | |
|---|---|---|---|---|
| | Female | Male | Female | Male |
| 40–44 | 4.1 | 8.3 | 82 407 | 46 300 |
| 45–49 | 8.6 | 18.1 | 43 519 | 24 074 |
| 50–54 | 13.1 | 28.9 | 32 407 | 17 593 |
| 55–59 | 22.6 | 68.6 | 21 296 | 8148 |
| 60–64 | 31.3 | 140.4 | 17 592 | 4352 |
| 65–69 | 53 | 242.2 | 12 037 | 2685 |
| 70–74 | 83.4 | 347.2 | 9259 | 1944 |

### ACKNOWLEDGEMENTS

We thank Kazuhisa Hanamura and Kazuhiro Asakura, former researchers at the Transmission Advancement Organisation of Japan Matsumoto Research Centre for their contribution in designing and conducting the CT screening for lung cancer. Masaomi Takizawa was a co-investigator who assisted in the design of the study. Yoshiro Takahashi and Tadashi Minemura co-operated in conducting the study. We thank the staff of Anti-Tuberculosis Association, Nagano Prefecture Branch, for their co-operation in implementing this programme. This study was carried out as a research programme of the Matsumoto Research Centre of the Transmission Advancement Organisation of Japan, which was established between 1995–1999.

© 2001 Cancer Research Campaign

### REFERENCES

Eddy DM (1981) The economics of cancer prevention and detection: Getting more or less. *Cancer* **47**: 1200–1209

Flehinger BJ, Kimmel M, Polyak T and Melamed MR (1993) Screening for lung cancer. The Mayo lung cancer project revisited. *Cancer* **72**: 1573–1580

Henschke CI, Miettinen OS, Yankelevitz DF, Libby DM and Smith JP (1994) Radiographic screening for cancer. Proposed paradigm for requisite research. *Clinical Imaging* **18**: 16–20

Henschke CI, MacCauley DI, Yankelevitz DF, Naidich DP, McGuinness, Miettinen OS, Libby DM, Pasmantier MW, Koizumi J, Altorki NK and Smith JP (1999) Early lung cancer action project: Overall design and findings from baseline screening. *Lancet* **354**: 99–105

Kaneko M, Eguchi K, Ohmatsu H, Kakinuma R, Naruke T, Suemasu K and Moriyama N (1996) Peripheral lung cancer: screening and detection with low-dose spiral CT versus radiography. *Radiology* **201**: 798–802

Landis SH, Murray T, Bolden S and Wingo PA (1998) Cancer Statistics. California *Cancer J Clin* **48**: 6–29

Mountain CF (1997) Revisions in the international system for staging lung cancer. *Chest* **111**: 1710–1717.

Nishizawa K, Iwai K, Matsumoto T, Sakashita K, Iinuma TA, Tateno Y, Miyamoto T, Shimura A and Takagi H (1996) Estimation of the exposure and a risk-benefit analysis for a CT system designed for a lung cancer mass screening unit. *Radiation Protection Dosimetry* **67**: 101–108

Oda M, Watanabe Y, Shimizu J, Murakami S, Ohta Y, Sekido N, Watanabe S, Ishikawa N and Nonomura A (1998) Extent of mediastinal node metastasis in clinical stage 1 non-small-cell lung cancer: The role of systematic nodal dissection. *Lung Cancer* **22**: 23–30

Sagawa M, Saito Y, Takahashi S, Takahashi S, Usuda K, Kamma K, Sato M, Ota S, Nagamoto N, Fujimura S, Nakada N, Hashimoto K, Suda H, Imai T and Saito H (1990) Clinical and prognostic assessment of patients with resected small peripheral lung cancer lesions. *Cancer* **66**: 2653–2657

Soda H, Tomita H, Kohno S and Oka M (1993) Limitation of annual screening chest radiography for the diagnosis of lung cancer. *Cancer* **72**: 2341–2346

Sone S, Takashima S, Li F, Yang Z-G, Honda T, Maruyama Y, Hasegawa M, Yamanda T, Kubo K, Hanamura K and Asakura K (1998) Mass screening for lung cancer with a mobile spiral computed tomography scanner. *Lancet* **351**: 1242–1245

Sone S, Li F, Yang Z-G, Takashima S, Maruyama Y, Hasegawa M, Wang JC, Kawakami S and Honda T (2000) Characteristics of small lung cancers invisible on conventional chest radiography and detected by population-based screening using spiral CT. *Br J Radiol* **73**: 137–145

*© 2001 Cancer Research Campaign*
